# Current Advances in the Combination of Fatty Acids and Resveratrol to Fight Ocular Diseases

**DOI:** 10.1002/mnfr.70037

**Published:** 2025-04-07

**Authors:** Dominique Delmas, Maude Perus, Virginie Aires, François Hermetet

**Affiliations:** ^1^ Université Bourgogne Europe Dijon France; ^2^ INSERM Research Center UMR1231 ‐ Therapies and Immune Response in Cancers Team Bioactive Molecules and Health Research Group Dijon France; ^3^ Centre de Lutte Contre le Cancer Georges François Leclerc Dijon France; ^4^ INSERM UMS58 Biologie Santé Dijon BioSanD Dijon France

**Keywords:** AMD, angiogenesis, detergent‐resistant membranes, omega‐3 fatty acids, oxidative stress, polyphenols, resveratrol, VEGF, VEGF‐receptor

## Abstract

Omega‐3 polyunsaturated fatty acids (PUFA) and polyphenols have attracted interest to counteract ocular diseases and more specifically age‐related macular degeneration (AMD). This eye disease, which is the leading cause of irreversible blindness in industrialized countries, is characterized by damage to the central part of the retina, the macula. Despite therapeutic advances with the use of anti‐vascular endothelial growth factor (VEGF) monoclonal antibodies, numerous resistance mechanisms that worsen visual impairment have been identified. In this context, we highlight the exceptional potential of polyphenols and PUFA in addressing AMD through their actions on the different molecular mechanisms involved in AMD progression. More specifically, this review focuses on the current understanding of the effects of resveratrol, as well as docosahexaenoic and eicosapentaenoic acids, two prominent omega‐3 PUFA, and the combination of these compounds. We also discuss the limitations and explore future directions for the combined use of these natural products as preventive or complementary therapies to preserve vision and slow disease progression.

## Graphical abstract

Graphical abstract illustrating the effects of Resvega administration on choroidal neovascularization and resveratrol metabolism in the retina. The diagram illustrates that oral administration of Resvega leads to a decrease in choroidal neovascularization. Multiple resveratrol metabolites, including dihydro‐resveratrol and resveratrol‐3‐O‐glucuronide, are detected in the retina following Resvega intake. This suggests a potential mechanism by which Resvega may exert its therapeutic effects on retinal disorders associated with abnormal blood vessel growth

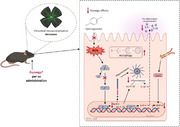

## 1 Introduction

**FIGURE 1:**
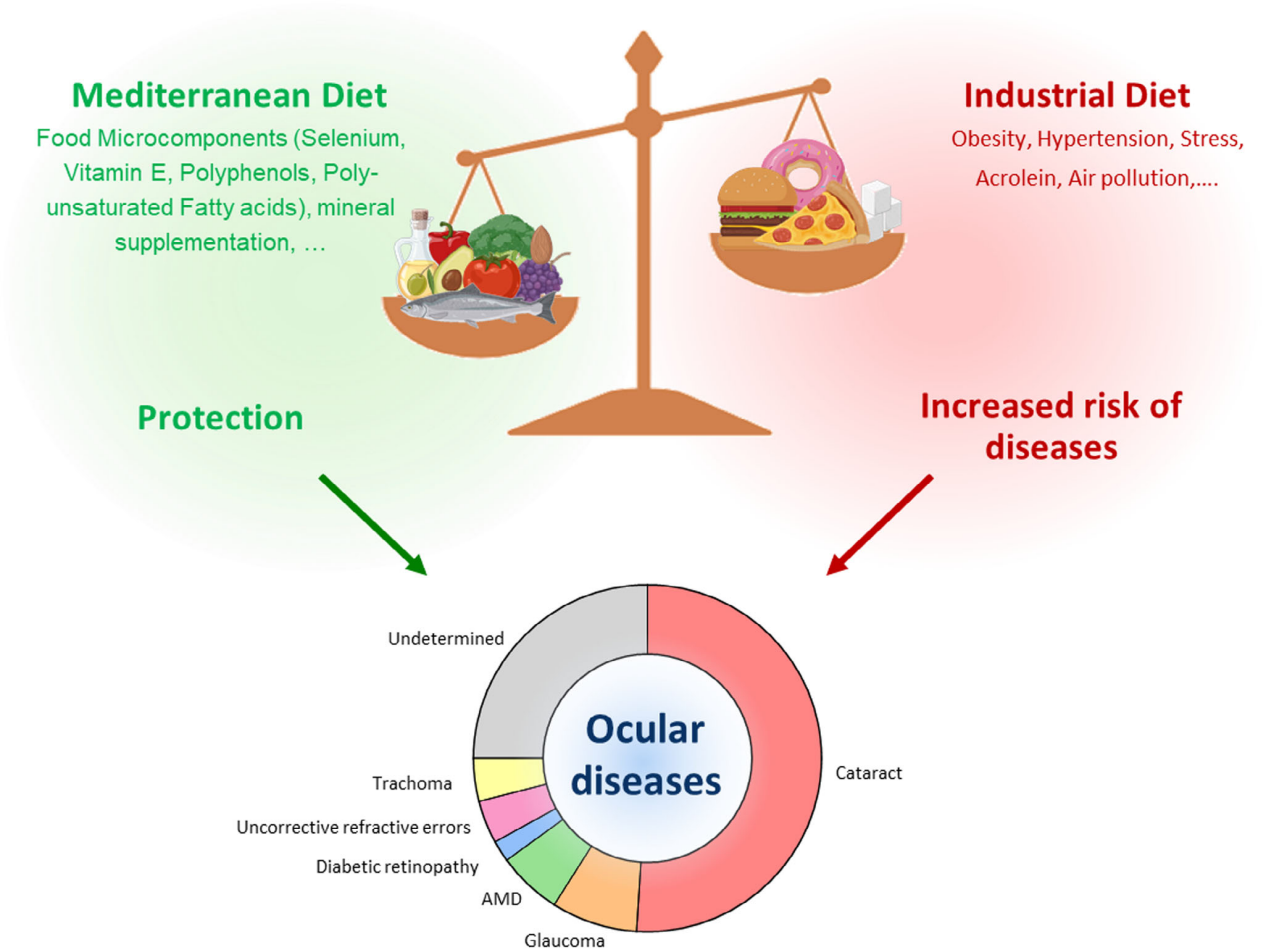
The benefits of a Mediterranean diet on eye diseases. A Mediterranean diet composed of foods rich in n‐3 FA (oily fish, olive oil, nuts, etc.) has a protective effect on various eye diseases, compared with an industrial diet rich in saturated FA and sugar.

Numerous epidemiological studies have highlighted the significant impact of environmental factors on human health. Adverse conditions, such as exposure to air pollution, poor dietary habits, and the prevalence of metabolic diseases, including obesity, hypertension, and dyslipidemia, have been identified as major contributors to the development of chronic illnesses [1, 2, 3]. These include cardiovascular diseases, diabetes, and various forms of cancer. Airborne pollutants, for instance, are known to increase systemic inflammation and oxidative stress, both of which are critical pathways leading to these conditions (Figure 1). Similarly, metabolic disorders often create an internal environment of chronic low‐grade inflammation, exacerbating the risk of long‐term complications. Conversely, a nutrient‐rich diet emphasizing green vegetables, fresh fruits, dietary fibers, and foods containing bioactive compounds, such as antioxidants, has been shown to reduce the probability of developing these debilitating pathologies [4, 5, 6]. Antioxidants, for instance, combat oxidative stress by neutralizing free radicals, which are key drivers of cellular damage and disease progression. Incorporating these foods into one's diet not only enhances overall health but also provides protective effects against many chronic diseases (Figure 1). In a parallel manner, the same harmful environmental factors, such as oxidative stress and inflammation induced by air pollution or metabolic imbalances, are increasingly recognized as contributing to the onset and progression of ocular diseases. For example, prolonged exposure to high levels of oxidative stress can damage the delicate tissues of the eye, leading to conditions such as cataracts, glaucoma, or age‐related macular degeneration (AMD). On the other hand, protective dietary elements such as vitamin E, polyunsaturated fatty acids (PUFA), and polyphenols have demonstrated potential in reducing the risk of these ocular pathologies. Vitamin E, a potent antioxidant, helps to prevent cellular damage in retinal tissues, while PUFA, particularly omega‐3 fatty acids (n‐3 FA), have anti‐inflammatory properties that support eye health. Polyphenols, found in foods like berries, tea, and red wine, exhibit a range of biological activities, including antioxidant, anti‐inflammatory, and anti‐angiogenic effects, making them promising candidates for ocular protection.

Focusing specifically on AMD, one of the leading causes of irreversible blindness in aging populations, we describe in this review how polyphenols and PUFA stand out as particularly promising [7]. Indeed, AMD is closely associated with processes such as chronic inflammation, oxidative stress, and pathological angiogenesis. By mitigating these mechanisms, polyphenols and PUFA could play a crucial role in preserving vision and slowing down the progression of disease. Their potential as preventive or adjunct therapies is supported by increasing evidence from preclinical and clinical studies, indicating that these compounds may help alleviate the burden of AMD and improve the quality of life for those affected.

This review focuses on the current understanding of the effect of resveratrol (RSV), an emblematic polyphenol, along with docosahexaenoic acid (DHA) and eicosapentaenoic acid (EPA), two prominent n‐3 FA.

## 2 AMD: A Multifaceted Eye Disease With a Pivotal Role for VEGF Signaling

AMD is the primary cause of vision loss among individuals over the age of 65 in developed countries. It is an irreversible degenerative condition affecting the retina, specifically targeting the macula, which is the central region responsible for sharp, detailed vision. The gradual deterioration of the macula leads to a progressive loss of central vision, often manifesting as a blurred spot in the center of the visual field (scotoma), distortion of straight lines (metamorphopsia), or in advanced stages, a complete darkening of central vision. While peripheral vision is typically preserved, the impairment of tasks like reading, recognizing faces, and driving significantly reduces quality of life.

**FIGURE 2:**
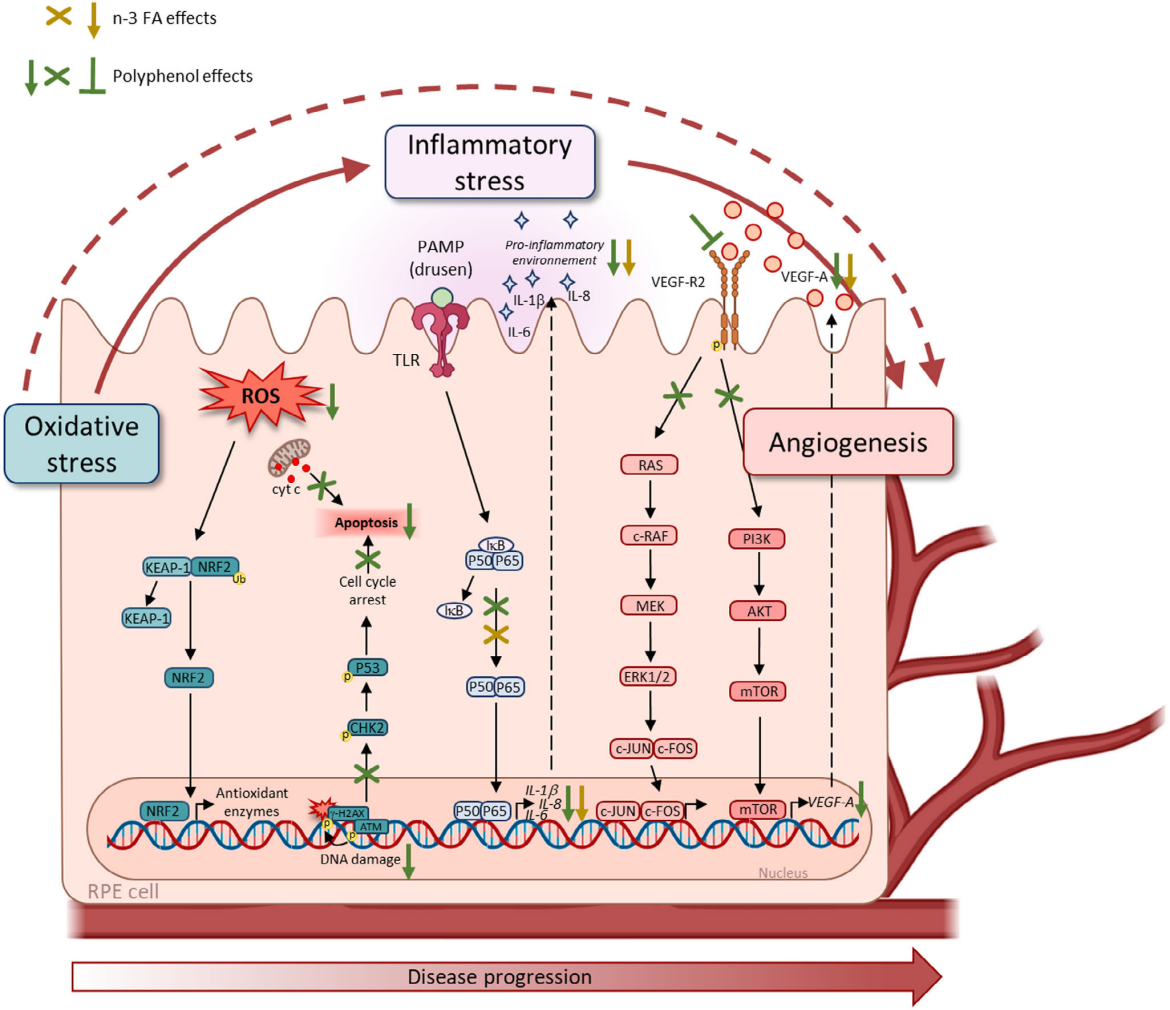
The effects of polyphenols and n‐3 FA on cellular mechanisms triggered in RPE cells. Oxidative stress is one of the first mechanisms triggered during the development of AMD in RPE cells. ROS promote the release of the transcription factor NRF2, which migrates into the nucleus to induce the transcription of antioxidant enzyme genes. In the presence of polyphenols (green arrows), this mechanism is enhanced to boost the cell's antioxidant defenses. Polyphenols will also reduce DNA damage, identified via histone H2AX phosphorylation, and the DNA damage response pathway (DDR pathway). These polyphenols will also protect against mitochondrial damage, thus protecting cells from apoptosis. After the onset of oxidative stress, inflammatory stress is set in place. n‐3 FA (brown arrows) and polyphenols have the ability to protect against inflammatory stress by decreasing the activation of the NF‐kB pathway, ultimately decreasing the secretion of pro‐inflammatory cytokines. Then, angiogenesis is set in place. It is promoted by both oxidative stress and inflammatory stress. The binding of VEGF‐A to VEGF‐R2 is inhibited in the presence of polyphenols such as RSV. This results in a decrease in the signaling pathways associated with the synthesis of VEGF‐A. n‐3 FA and polyphenols both have the ability to decrease the secretion of VEGF‐A.

AMD is commonly classified into two forms as follows: dry AMD (atrophic) and wet AMD (neovascular or exudative). Dry AMD is the more prevalent form, accounting for approximately 80%–90% of cases. It is characterized by the accumulation of drusen, yellowish extracellular deposits, between the retinal pigment epithelium (RPE) and Bruch's membrane. These deposits disrupt the normal function of the RPE and photoreceptors, leading to their gradual degeneration. While dry AMD progresses slowly, it lacks effective therapeutic options beyond lifestyle modifications and dietary supplementation [8]. Besides dry AMD form, wet AMD, though less common, accounts for the majority of severe vision loss associated with the condition. It is characterized by abnormal blood vessel growth (choroidal neovascularization) originating from the choroid (Figure 2). These vessels invade the retina, leaking fluid or blood and causing rapid and severe damage to the macula. The pathological angiogenesis is driven by the vascular endothelial growth factor (VEGF), a key signaling protein. Actually, some therapies are used in wet AMD, to inhibit neovascularization, primarily through two approaches as follows: (i) laser photocoagulation, an older technique where laser energy is used to destroy abnormal blood vessels. However, this method is non‐selective and often damages surrounding healthy tissue, resulting in scarring and further vision loss; (ii) anti‐VEGF therapy: intravitreal injections of VEGF inhibitors, such as ranibizumab, bevacizumab, or aflibercept, are now the gold standard [9, 10, 11, 12, 13, 14]. These agents block VEGF‐A from binding to its receptor, preventing the growth of new blood vessels and reducing vascular permeability. Despite their effectiveness in preserving vision, these treatments require repeated injections and are associated with risks such as inflammation, increased intraocular pressure, bleeding, and, in rare cases, endophthalmitis [15, 16]. Concerning dry AMD, no approved therapies currently halt or reverse the disease. Nutritional supplementation, based on findings from the Age‐Related Eye Disease Studies (AREDS1 and AREDS2), remains the only widely recommended intervention [17, 18, 19, 20]. These supplements, which include vitamins C and E, zinc, copper, lutein, and zeaxanthin, have been shown to slow the progression of intermediate AMD to advanced stages, but they do not restore lost vision or address underlying mechanisms.

## 3 Potential Interest of Omega‐3 Fatty Acids and Resveratrol in AMD

Given the limitations of current therapies, there is an urgent need for novel approaches to address both forms of AMD. n‐3 FA and RSV are emerging as promising candidates due to their multifaceted biological activities.

### 3.1 Omega‐3 Fatty Acids

Found in fish oils and certain plant sources, these PUFA, particularly in n‐3 PUFA such as EPA and DHA, have anti‐inflammatory, anti‐angiogenic, and neuroprotective properties. In physiological conditions, the retina is very rich in n‐3 PUFA, especially DHA which represents more than 50% of FA of the external segments of the photoreceptors [21]. It also plays a crucial role in the functioning of the retina. DHA is essential for visual transduction. It is incorporated into the membranes of the retinal cells and plays a major role in the conversion of the light signal into electrical signals that ultimately reach the brain [22]. DHA increases the activity of lysosomal acid lipase, an enzyme that helps remove “waste” from the retina. The outer segments are digested by the RPE and excreted into the bloodstream. The structure of photoreceptors and their function are affected by the biophysical and biochemical properties of DHA. In addition, phospholipid FA are the primary sources of signaling molecules that modulate intercellular communication and autocrine signals from plasma membranes. To enable proper physiological function and renewal, an adequate supply of DHA is therefore necessary. This is especially true in pathological conditions such as AMD where it is important to preserve the patients’ central vision and ensure proper photoreceptor function. Indeed, a depletion of DHA impacts the structure of the membrane of the external segments of the photoreceptors that disrupts the organization of rhodopsin (the primary light‐sensitive receptor protein in rod photoreceptor cells of the retina). EPA and DHA are thought to reduce retinal inflammation and oxidative stress, both of which are critical in AMD pathogenesis. They reduce the production of pro‐inflammatory cytokines and promote the synthesis of specialized pro‐resolving mediators, such as resolvins and protectins, as shown in cardiovascular diseases [23]. These properties could help to resolve chronic inflammation in the retina since chronic inflammation is a key driver of AMD progression, particularly in the presence of oxidative stress [24]. Several studies have been able to identify an association between a sufficient dietary intake of n‐3 FA as DHA or fish oils and a reduction in the occurrence of neovascular AMD. A notable meta‐analysis conducted by Chong et al. reviewed data from nine studies and found that individuals with the highest levels of dietary n‐3 FA intake had a 38% lower risk of late AMD compared to those with the lowest intake. Similarly, higher fish consumption (at least two servings per week) was associated with a 24% reduced risk of early AMD [25]. Another comprehensive review by SanGiovanni and Chew highlighted the neuroprotective and anti‐inflammatory roles of DHA and EPA, emphasizing their capacity to modulate retinal inflammation and reduce the expression of VEGF, a key driver of wet AMD [26]. An Australian study (“Blue Mountains Eye Study”) showed that regular consumption of fish, even in small quantities, was associated with a significant reduction in the risk of developing late AMD [27]. Another multicenter American study involving 349 people with AMD and 504 controls shows an increase in the risk of AMD with the consumption of vegetable fats, the consumption of monounsaturated FA, and the consumption of omega‐6 FA. On the other hand, a high consumption of n‐3 FA reduces the risk of AMD. This effect is more marked when the consumption of omega‐6 FA is low [28]. Conversely, in a study of 567 individuals with AMD, total fat or linolenic acid consumption was found to be correlated with an increased risk of AMD, while DHA consumption had a protective effect. Cho et al. have highlighted that a high consumption of fish (canned tuna, dark‐fleshed fish [mackerel, swordfish, salmon, sardines, bluefish], and white‐fleshed fish) per week reduced the risk by 35% [29]. A first French study (Nutritional AMD Treatment 1 [NAT1]) showed that AMD lesions were stabilized in patients supplemented with DHA. The progression of the disease could then be slowed by correcting n‐3 FA deficiencies [30]. Recently a cross‐sectional study utilizing data from the National Health and Nutrition Examination Survey (NHANES), including 4842 participants aged 40 years and older, revealed that higher dietary EPA and DHA intake could be associated with lower AMD risk in the older US population [31]. The mechanisms of action of DHA are multiple. Indeed, it could act on mitochondrial activity by preserving membrane integrity, thereby preventing cell apoptosis and protecting cells from oxidative stress [32]. As previously mentioned, the structure of photoreceptors as well as their function are affected by the biophysical and biochemical properties of DHA. In addition, phospholipid FA are the primary sources of signaling molecules that modulate intercellular communication and autocrine signals from plasma membranes. To allow proper physiological functioning and renewal, a sufficient supply of DHA is therefore necessary. This is especially relevant in pathological conditions such as AMD, where preserving patients’ central vision and ensuring proper photoreceptor function is crucial. In colorectal cancer, n‐3 FAs have been shown to reduce VEGF expression by inhibiting the COX‐2/PGE2 pathway, leading to the suppression of ERK1/2, a crucial component of VEGF synthesis [33, 34]. Further research is needed to fully elucidate this mechanism in retinal cells. Additionally, n‐3 FA may modulate VEGF expression, offering potential benefits for both wet and dry AMD.

### 3.2 Polyphenols

Polyphenols, a diverse class of phytochemicals found in fruits, vegetables, tea, coffee, and wine, have gained attention for their health benefits, especially in relation to age‐related diseases. These compounds include flavonoids, phenolic acids, lignans, and stilbenes, all of which exhibit potent antioxidant and anti‐inflammatory properties (Figure 2). Given the role of oxidative stress and chronic inflammation in AMD pathogenesis, polyphenols offer a promising therapeutic strategy to address different stages of AMD progression. Indeed, polyphenols can counteract the initial event of oxidative damage by neutralizing reactive oxygen species (ROS) and reduce oxidative damage, which is crucial in AMD pathology.

Research highlights specific polyphenolic compounds, including RSV, quercetin, and catechins, for their potent antioxidant properties. These compounds effectively scavenge free radicals and protect RPE cells from oxidative stress‐induced apoptosis, potentially offering therapeutic benefits in AMD. Epigallocatechin gallate (EGCG), a catechin in green tea, reduces ROS production and maintains mitochondrial function in photoreceptor cells [14, 35]. Another polyphenol, quercetin, present in apples and onions, protects RPE cells from oxidative damage by increasing glutathione levels and reducing apoptosis [36]. These antioxidant mechanisms could help preserve retinal cell integrity and potentially slow down AMD progression. Additionally, polyphenols address the crucial inflammatory component of AMD pathogenesis. Compounds such as curcumin and various flavonoids modulate key inflammatory pathways by inhibiting nuclear factor kappa B (NF‐κB) and cyclooxygenase‐2 (COX‐2) [37].

#### 3.2.1 Focus on Resveratrol

RSV, known for its antioxidant properties, can protect ocular tissues from oxidative stress. RPE cells, vital for retinal health, are particularly vulnerable to oxidative stress linked to AMD [38, 39]. Like other antioxidants such as vitamins C, E, and carotenoids, RSV may lower AMD risk by mitigating oxidative damage. Research demonstrates that RSV reduces ROS in RPE cells under both normal and stressed conditions, such as exposure to hydrogen peroxide. This antioxidant effect helps delay cell death and maintain cellular function, potentially offering protective benefits in the context of AMD [40]. The mechanism likely involves modulation of oxidative stress pathways, including inhibition of mitogen‐activated protein kinases (MAPK) and enhancement of antioxidant enzyme activity. These actions collectively contribute to RSV's potential as a therapeutic agent in retinal disorders characterized by oxidative stress [40]. RSV also protects against oxidative damage from environmental stressors like cigarette smoke and ultra‐violet (UV) radiation. Studies highlight its ability to shield RPE cells from acrolein and UV‐A‐induced damage by reducing ROS production and inflammatory responses [41, 42]. Additionally, RSV enhances mitochondrial bioenergetics and upregulates antioxidant defenses, thereby improving cell survival under stress conditions. Metabolic disorders such as diabetes and obesity exacerbate oxidative stress, negatively impacting ocular health. RSV has demonstrated multiple beneficial effects, including improved blood glucose management, enhanced mitochondrial function, and reduced inflammation. These systemic benefits may indirectly protect against AMD progression by mitigating key risk factors [43, 44]. Furthermore, RSV promotes autophagy, a process crucial for clearing cellular debris such as lipofuscin, which is a marker of aging and AMD progression [45]. This autophagy‐enhancing effect of RSV involves the inhibition of the mTOR‐ULK1 pathway, leading to increased formation of autophagosomes and improved cellular waste removal [46]. By facilitating the clearance of lipofuscin and other cellular debris, RSV may help mitigate the accumulation of harmful substances associated with AMD development and progression. This could also be achieved through mechanisms involving SIRT1 activation and enhanced proteasomal activity. RSV's anti‐inflammatory properties further contribute to AMD management by reducing levels of pro‐inflammatory cytokines and chemokines implicated in AMD‐related inflammation, including interleukin‐6 (IL‐6), interleukin‐8 (IL‐8), and C‐X‐C motif chemokine 11 (CXCL11) [47, 48]. Activation of peroxisome proliferator‐activated receptors (PPAR) pathways by RSV also protects RPE cells from oxidative and inflammatory injuries. In advanced stages of AMD, characterized by neovascularization, RSV may counteract angiogenesis by inhibiting VEGF secretion [49]. Studies indicate that RSV reduces VEGF levels induced by oxysterols and diabetes, highlighting its potential to mitigate neovascular complications. Preclinical research shows RSV can suppress inflammatory and angiogenic signals, decrease choroidal neovascularization, and maintain AMP‐activated protein kinase (AMPK) levels, thereby preventing NFκB activation in RPE–choroid complexes [50]. Recent clinical studies have explored the potential of RSV and quercetin, two polyphenols known for their anti‐inflammatory and antioxidant properties, in reducing neovascularization associated with AMD. In a noteworthy study conducted in octogenarian patients in the United States, oral administration of a combination of RSV and quercetin showed promising results in managing AMD symptoms [51, 52]. The trial demonstrated that this supplementation could mitigate neovascularization, leading to objective improvements in retinal health and visual function. These results were comparable to those achieved with anti‐VEGF therapies but with potentially fewer side effects. This outcome underscores the potential of polyphenol‐based strategies as a complementary or alternative approach to current AMD treatments (https://ichgcp.net/clinical‐trials‐registry/NCT05062486).

## 4 n‐3 PUFA and Resveratrol: A Promising Combination to Combat AMD

An important question is whether a combination of n‐3 FA and RSV could synergistically combat AMD. To address this, we have tested the potential effects of a formulation commercialized by Laboratoires Théa (Clermont‐Ferrand, France), Resvega which contains 30 mg of RSV and 665 mg of n‐3 FA, specifically 350 mg of EPA and 190 mg of DHA. To evaluate the potential anti‐angiogenic effects of Resvega, we employed a laser‐induced choroidal neovascularization (CNV) mouse model. Mice were supplemented for 14 days with Resvega, RSV alone, or Nutrof (containing only omega‐3 FA without RSV). The dosage used was 6 mg/kg of equivalent RSV, calculated based on the recommended human dose of two pills totaling 60 mg of RSV and 1.33 g of n‐3 FA daily [53].

**FIGURE 3:**
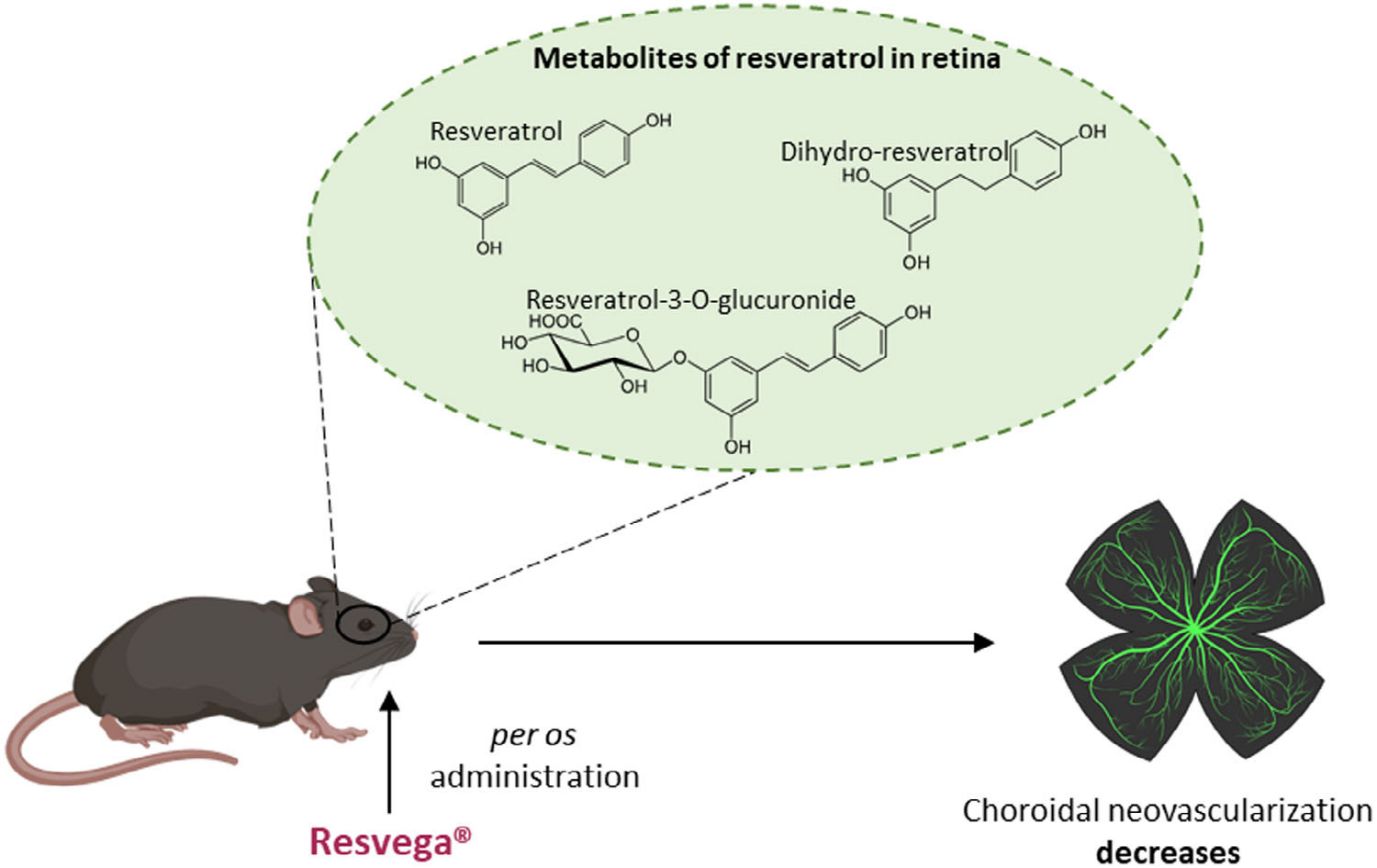
Resvega reduces choroidal neovascularization in mice. Mice impacted by laser to induce choroidal neovascularization (CNV) received per os administration of Resvega. Different metabolites of RSV were found in the retina: dihydro‐RSV and RSV‐3‐O‐glucoronide. Retinas from Resvega‐treated mice showed reduced CNV compared with untreated mice.

**FIGURE 4:**
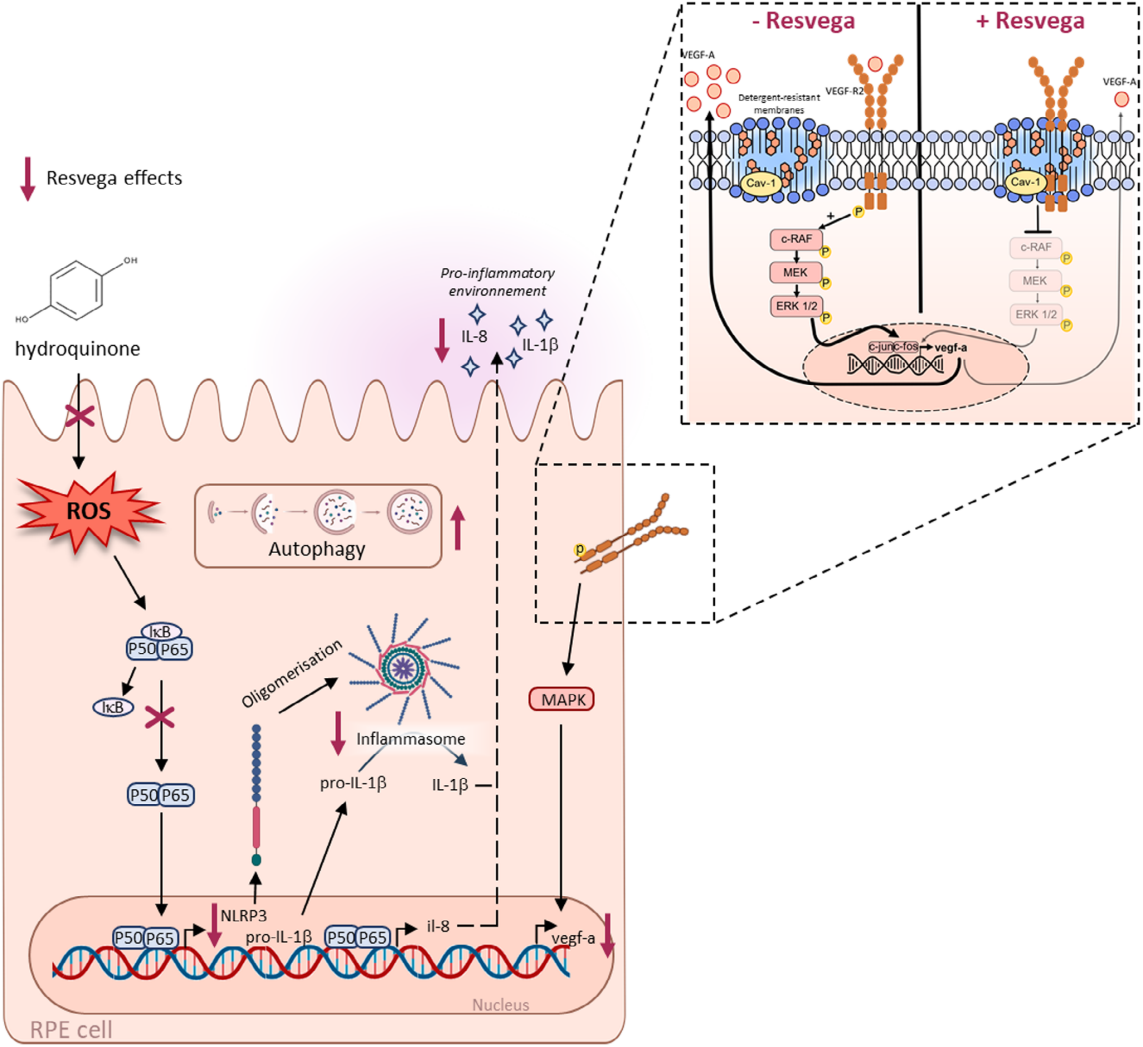
The identified mechanisms of Resvega in RPE cells. Hydroquinone‐induced ROS are captured by Resvega. It also reduces activation of the NF‐kB pathway, which enables transcription of NLRP3, which then oligomerizes to form the inflammasome. Pro‐IL‐1β is cleaved into IL‐1β by the inflammasome, helping to establish and maintain inflammatory stress in RPE cells. Resvega is able to reduce the inflammasome in RPE cells, thereby reducing inflammatory stress. Autophagy helps maintain retinal cell homeostasis. This cellular mechanism, altered in RPE cells, is restored in the presence of Resvega. Moreover, this formulation acts on VEGF‐A secretion. It induces interaction between VEGF‐R2 and CAV‐1 within detergent‐resistant membranes. VEGF‐R2 becomes inactive and the downstream signaling pathway is diminished, as is VEGF‐A synthesis and secretion.

On Day 0 (after 14 days of pre‐supplementation), four groups of mice were subjected to four laser applications on one eye. They then continued to receive their daily dose of nutraceuticals for an additional 21 days. Fluorescein angiography at the inner retinal level did not reveal significant differences between the supplemented groups compared to untreated mice. However, indocyanine green angiography highlighted that mouse supplemented with Resvega showed a significant reduction in choroidal neovascularization (CNV) development compared to both the control group and the Nutrof‐supplemented group (Figure 3). These initial results were corroborated by a global proteome analysis of the lasered retinas. The analysis revealed that Resvega induced a specific enrichment in the negative regulation of epithelial cell migration cluster in the retinas of supplemented mice. This included negative regulation of vasculature and blood vessel development, angiogenesis, blood vessel morphogenesis, and epithelial cell proliferation [53]. The molecular mechanism by which Resvega inhibits CNV and angiogenesis induction, appears to involve significant disruption of dynamic membranes, particularly the localization of VEGF receptors (VEGF‐R) in detergent‐resistant membranes (Figure 4). We have indeed demonstrated that Resvega, which exhibits superior effects compared to RSV or n‐3 FA used alone, can delocalize VEGF‐R2 into detergent‐resistant membranes, promoting strong interactions with caveolin‐1 (CAV‐1), a key protein in these membrane structures [54]. This specific interaction, confirmed by various methods including microscale thermophoresis, co‐immunoprecipitation, and proximity ligation assays, is essential for the signaling cascade that produces VEGF in human retinal cells [54]. Disruption of detergent‐resistant membranes using a cholesterol‐chelating agent, such as methyl‐β‐cyclodextrin, impairs the interaction between VEGF‐R2 and CAV‐1. Consequently, this disruption diminishes the effectiveness of Resvega in reducing VEGF‐R2 activation and the subsequent activation of mitogen‐activated protein (MAP) kinases, including Raf, MEK, and ERK proteins [55]. Very interestingly, the Resvega formulation was effective in preventing the nuclear delocalization of transcription factors, particularly c‐JUN. DNA chip array analyses indicate that Resvega inhibits the binding of this nuclear factor to response elements that regulate the transcription of VEGF‐R and VEGF genes, ultimately leading to a reduction in VEGF production [55].

RSV employs multiple mechanisms to limit VEGF production in retinal cells. Notably, a 2022 study by Sghaier et al. highlights Resvega's ability to disrupt VEGF‐A secretion by modulating the PI3K‐AKT‐mTOR and NF‐κB signaling pathways (Figure 4). Furthermore, Resvega interferes with the multimolecular complex known as IκB kinase (IKK), significantly reducing the levels of both IKKα and IKKβ, as well as their phosphorylated forms, along with IKKγ [56]. This disruption of the IKK complex is crucial because it plays a central role in the phosphorylation of IκB proteins, which normally sequester NF‐κB dimers in the cytoplasm. By inhibiting this process, Resvega enhances the cytosolic retention of IκB and prevents the translocation of NF‐κB subunits to the nucleus, thereby reducing their ability to stimulate the transcription of target genes such as VEGF‐A. Such a mechanism of action has also been described for bevacizumab, a widely used anti‐VEGF therapy [56]. Overall, these findings suggest that Resvega not only limits VEGF production through direct interaction with key signaling pathways but also alters the dynamics of transcription factor localization, contributing to its potential as a therapeutic agent in managing AMD (Figure 4).

Other mechanisms significantly impact the progression of AMD, particularly the impairment of autophagic and proteasomal clearance mechanisms in RPE (Figure 4). In this context, Resvega has demonstrated the ability to induce autophagy and enhance the survival of ARPE‐19 cells under conditions of proteasome inhibition [57]. Resvega increased autolysosome formation, autophagic flux, and modulated p62 and LC3 protein levels, which were confirmed by fluorescent microscopy, providing cytoprotection in proteasome‐inhibited cells [57]. These results underscore the potential of Resvega and similar nutraceuticals in preventing damage to ARPE‐19 cells. Studies also indicate that RSV, whether used alone or in combination with n‐3 FA, as in Resvega, enhances the viability of RPE cells exposed to hydroquinone, a compound derived from cigarette smoke. This formulation has been shown to reduce inflammatory markers, including interleukin‐8 (IL‐8) and monocytic chemoattractant protein‐1 (MCP‐1), thereby alleviating stress caused by hydroquinone and improving impaired protein clearance [58, 59].

In another pathology, such as diabetic retinopathy (DR), Resvega has demonstrated the ability to reverse retinal microvascular alterations. Preclinical findings, supported by advanced imaging techniques like optical coherence tomography angiography (OCTA) and adaptive optics (AO) ophthalmoscopy, indicate its potential for future clinical trials in DR. Resvega also improved cell membrane integrity in RPE cells by reducing lactate dehydrogenase (LDH) leakage. Additionally, it diminished caspase‐1 activity, NLRP3 inflammasome release, and the secretion of interleukin‐1 beta (IL‐1β) and IL‐8 in IL‐1α‐primed ARPE‐19 cells [60]. This is particularly significant because NLRP3 inflammasome activation remains a critical target for managing and treating AMD [61]. Resvega has the potential to reduce NLRP3 inflammasome‐mediated inflammation in RPE cells with impaired protein clearance [62].

Furthermore, Resvega, in conjunction with antioxidants such as N‐acetylcysteine (NAC) and aminopyrrolidine‐2,4‐dicarboxylic acid (APDC), effectively alleviated oxidative stress induced by hydroquinone in RPE cells by suppressing NADPH oxidase activity (Figure 4). These findings collectively demonstrate that hydroquinone exacerbates cytotoxicity and oxidative stress in RPE cells, while RSV‐based formulations like Resvega can mitigate these harmful effects, underscoring their potential in the management of AMD and related conditions [58].

## 5 Limitations and Future Perspectives for Resvega Use

While these findings are encouraging, several limitations need to be addressed. Indeed, most studies on Resvega have been conducted in vitro or in animal models. Translating these results into clinical efficacy in human patients requires robust clinical trials. Translating preclinical findings into clinical practice presents significant challenges. Key obstacles include determining optimal dosage, ensuring bioavailability of active compounds in retinal tissues, and accounting for inter‐individual variability in treatment response. Robust, well‐designed clinical trials are essential to validate the efficacy and safety of Resvega across diverse patient populations. Moreover, long‐term studies are needed to assess potential side effects and therapeutic benefits in patients with chronic ocular conditions like AMD. Moreover, the most effective delivery method to target retinal cells remains unclear. The bioavailability of Resvega's active compounds and their ability to reach retinal tissues in sufficient concentrations require further exploration especially RSV metabolites or n‐3 FA metabolites. Actually, the long‐term effects of Resvega, especially when used alongside other therapeutic agents, need thorough evaluation to ensure safety and sustained benefits. Finally, variability in genetic, dietary, and environmental factors could influence Resvega's efficacy, necessitating studies across diverse populations.

To advance the use of Resvega in ocular health, future research should focus on the following:

‐ Clinical Trials: Large‐scale, randomized clinical trials are essential to validate Resvega's efficacy and safety in patients with AMD and other retinal disorders;
‐ Combination Therapies: Investigating Resvega as an adjunct to existing therapies, such as anti‐VEGF treatments, could provide synergistic benefits and reduce treatment burdens;
‐ Personalized Medicine: Identifying biomarkers to predict patient responses to Resvega could help tailor interventions for maximum efficacy;
‐ Innovative Delivery Systems: Recent advancements in nanotechnology offer promising strategies for ocular drug delivery. Gold nanoparticles have shown potential to increase drug residence time on the ocular surface, potentially reducing administration frequency for various eye treatments [63]. Polymer‐based nanoparticles, such as conjugated polymer nanoparticles (P3HT‐NP), have demonstrated efficacy in restoring vision in animal models of retinal degeneration, with implications for age‐related macular degeneration (AMD) treatment [64]. Additionally, non‐spherical nanoparticles, in combination with iontophoresis, are being explored to enhance ocular drug delivery and overcome the limitations of traditional eye drops and intravitreal injections [65]. A novel “light scalpel” technology utilizing gold nanoparticles and femtosecond lasers has also been developed for targeted drug and gene delivery to specific areas of the eye, showing potential for treating glaucoma, retinitis, and macular degeneration [66];
‐ Expanded Applications: Beyond AMD, Resvega's anti‐inflammatory and antioxidant properties could be explored for other ocular conditions, such as glaucoma or uveitis, broadening its therapeutic scope.

## 6 Conclusion

Resvega, a nutraceutical combining *trans*‐RSV and n‐3 FA, has emerged as a promising candidate for managing ocular diseases like AMD. Its multifaceted mechanisms address key pathological processes such as oxidative stress, inflammation, angiogenesis, and impaired autophagy. By targeting these interconnected pathways, Resvega has shown potential to complement existing therapies and provide cytoprotective benefits in retinal cells under various stress conditions. However, its full potential will only be achieved through rigorous clinical validation and technological innovations in drug delivery. In this way, Resvega could offer a dual‐action strategy which complements existing therapies and may reduce the frequency or intensity of invasive treatments. These studies should reinforce the importance of nutraceuticals in addressing unmet needs in AMD management, paving the way for integrative therapeutic strategies.

## Conflicts of Interest

Neither the authors nor their institutions have financial or professional ties to Resvega.

## Data Availability

Data sharing not applicable to this article as no datasets were generated or analyzed during the current study
